# Platelet-To-Lymphocyte Ratio Efficiency in Predicting Major Adverse Cardiovascular Events After Percutaneous Coronary Intervention in Acute Coronary Syndromes: A Meta-Analysis

**DOI:** 10.31083/RCM27942

**Published:** 2025-05-21

**Authors:** He Wang, Tuerxun Zulikaier, Balati Yumaierjiang, Saiqi Lyu, Pengyi He

**Affiliations:** ^1^Clinical Medicine Department, Xinjiang Medical University, 830054 Urumqi, Xinjiang, China; ^2^Heart Center, The Fifth Affiliated Hospital of Xinjiang Medical University, 830011 Urumqi, Xinjiang, China

**Keywords:** platelet-to-lymphocyte ratio, acute coronary syndromes, percutaneous coronary intervention, major adverse cardiovascular events, meta-analysis

## Abstract

**Background:**

The platelet-to-lymphocyte ratio (PLR) is applied as a potential first-line prognostic predictor for many cardiovascular diseases due to its simplicity and accessibility. This meta-analysis aimed to quantify the predictive power of PLR for major adverse cardiovascular events (MACEs) in patients with acute coronary syndrome (ACS) undergoing percutaneous coronary intervention (PCI), explore its predictive efficacy in different populations, and identify other potential influencing factors.

**Methods:**

PubMed, Embase, Cochrane Library, and Web of Science databases were comprehensively searched for eligible studies until February 7, 2025, based on the inclusion and exclusion criteria. The Newcastle–Ottawa scale (NOS) was employed for quality assessment. Sensitivity, specificity, summary receiving operating characteristic (SROC) and area under the curve (AUC) were combined using Stata 15.1 and Meta-DiSc software. Meta-regression analyses, subgroup analyses, threshold effect analyses, sensitivity analyses, and publication bias tests were performed.

**Results:**

Nine studies (7174 patients) were enrolled. High PLR could predict MACEs in ACS patients undergoing PCI, with 0.68 sensitivity (95% CI, 0.60–0.76), 0.65 specificity (95% CI, 0.57–0.73), and 0.72 AUC (95% CI, 0.68–0.76). Subgroup analyses noted that PLR better predicted MACEs after PCI in ACS patients in the subgroup with a higher proportion of female patients and the subset aged >60 years. Meta-regression analyses unveiled that study type (*p* < 0.01) and PLR cutoff value (*p *< 0.01) might be sources of heterogeneity in the sensitivity analyses, while the mean age (*p* < 0.001) and sex ratio (*p* = 0.05) might be sources of heterogeneity in the specificity analyses.

**Conclusions:**

High PLR levels have favorable values in predicting in-hospital and long-term MACEs after PCI in ACS patients. The PLR had greater sensitivity and an improved ability to identify risk in patients aged >60 years and the subgroup with a higher proportion of women and was also more sensitive to in-hospital MACEs.

**The PROSPERO Registration:**

No. CRD42024537586, https://www.crd.york.ac.uk/PROSPERO/view/CRD42024537586.

## 1. Introduction

Acute coronary syndromes (ACS) are acute manifestations of ischemic heart disease, including ST-segment elevation myocardial infarction (STEMI), non-ST-segment elevation myocardial infarction (NSTEMI), and unstable angina pectoris [1]. The 2023 European Society of Cardiology (ESC) guidelines for the management of ACS state that ischemic heart disease is the chief cause of cardiovascular disease (CVD) deaths, accounting for 38% of all CVD deaths in females and 44% in males in ESC member countries [2]. It is estimated that more than 7 million people worldwide are diagnosed with ACS every year, and approximately 5% of ACS patients die before hospital discharge [2, 3, 4]. The age-standardized mortality rates for ACS are higher in both males and females in low- and middle-income countries than those in higher-income countries, which undoubtedly exacerbates the disease burden [3]. Percutaneous coronary intervention (PCI) is recognized as an effective modality for ACS [4]. However, ACS patients remain at high risk of recurrent ischemic events and major adverse cardiovascular events (MACEs) after PCI [5]. Related articles have reported [6, 7, 8, 9, 10] that ACS patients undergoing PCI are likely to experience adverse outcomes such as coronary revascularization, heart failure, death, myocardial infarction, or stroke. In addition, PCI may promote inflammatory responses that increase the risk of coronary thrombosis, in-stent restenosis, and unstable coronary plaques, thereby undermining the clinical benefits of PCI [11, 12, 13]. Therefore, early prediction and identification of prognosis after PCI in ACS patients is crucial.

Inflammation and thrombosis are key to the pathogenesis and progression of ACS. Leukocytes are the main inflammatory mediators, and platelets are indicative of the inflammatory state and can be activated in the pro-inflammatory and pro-thrombotic microenvironment, leading to thrombosis. Thus, both leukocytes and platelets are closely associated with adverse events after PCI [14]. In recent years, platelet-to-lymphocyte ratio (PLR) has been recognized as a potential inflammatory marker capable of predicting cardiac events with affordability and widespread accessibility. PLR measurement includes both inflammatory and thrombotic pathways and may offer greater prognostic power than individual platelet or lymphocyte counts [15]. However, there are potential challenges and controversies in its application as a clinical tool. Previous researches showed that the PLR had a strong predictive value for mortality and MACEs in patients with coronary artery disease (CAD) and was an independent risk factor for both early and late mortality in patients with diabetes and STEMI [16, 17]. However, Kazem* et al*. [16] indicated that PLR could only identify high-risk ACS patients over 65 years old with catastrophic events. Additionally, the predictive power of PLR may be influenced by the selected threshold and the patient baseline conditions (e.g., comorbidities).

The results of previous studies are controversial. Therefore, we conducted this meta-analysis to evaluate the predictive power of PLR in MACEs after PCI in ACS patients, explore its predictive efficacy in different populations, and identify other potential influencing factors. This paper aims to integrate the clinical practice and specific outcomes of PLR in predicting the prognosis of ACS patients receiving PCI, which may identify high-risk patients with poor prognosis and thus provide greater clinical benefits.

## 2. Literature Review

This paper followed the Preferred Reporting Items for Systematic reviews and Meta-Analyses (PRISMA) [17] guidelines. The study protocol was registered with the PROSPERO (https://www.crd.york.ac.uk/PROSPERO/view/CRD42024537586) (No. CRD42024537586).

### 2.1 Search Strategy

PubMed, Embase, Cochrane Library, and Web of Science databases were searched for related studies up to February 7, 2025, with the language restricted to English. A combination of subject and free words was used, and search terms included (a) “platelet”; (b) “lymphocyte”; (c) “acute coronary syndrome (ACS)”, “ST-segment elevation myocardial infarction (STEMI)”, “non-ST-segment elevation myocardial infarction (NSTEMI)”, “unstable angina (UA)”, and (d) “percutaneous coronary intervention (PCI)”. In addition, the reference lists of selected literature were manually searched to avoid missing potentially relevant studies. The search strategy is displayed in **Supplementary Table 1**.

### 2.2 Inclusion and Exclusion Criteria

Articles were included if they met the following criteria: (1) study population: ACS patients treated with PCI; (2) exposure: high PLR; (3) outcome: MACEs; (4) study type: cohort study; (5) providing relevant data for calculating sensitivity and specificity or area under the curve (AUC) values.

The exclusion criteria were as follows: (1) types of publications: literature reviews, meta-analyses, case reports, conference abstracts, responses, letters, comments, animal experiments, basic research, irrelevant study topics, incomplete data, and duplicates; (2) any previous coronary surgery (e.g., PCI, coronary artery bypass graft); (3) any condition that impacted platelet and lymphocyte levels, such as hematological disorders, cancers, severe kidney or liver diseases, infections, systemic inflammatory conditions, autoimmune disorders, and treatment with fibrinolytic agents (only or before referral for pPCI).

### 2.3 Data Extraction

Data were extracted independently by two reviewers, and any disagreement was tackled through discussion with a third researcher. Extracted data included first author, publication year, country, study type, sample size, mean age, sex ratio of patients, time of MACEs, follow-up duration, PLR cutoff value, sensitivity, specificity, true positive (TP), false positive (FP), false negative (FN), and true negative (TN).

### 2.4 Quality Assessment

Study quality was appraised using the Newcastle-Ottawa Scale (NOS) for cohort studies in three domains: (1) study population selection, (2) comparability, and (3) exposure or outcome evaluation. The NOS consists of eight items, with two to five possible answers to each question. One star was given to answers of high quality, and each item had a maximum of one star (up to two stars for comparability questions). Each study had a maximum of nine stars. Finally, each study was evaluated for quality based on stars earned. Research with seven or more stars was considered to have a low likelihood of bias and high quality [18, 19, 20].

### 2.5 Statistical Analysis

TN, TP, FP, and FN were statistically analyzed for each study using Stata 15.1 software (StataCorp LLC, Coolidge, TX, USA), and sensitivity, specificity, positive likelihood ratio (LR), negative LR, and diagnostic odds ratio (DOR) were fully assessed. Summary receiving operating characteristic (SROC) curves were constructed, and the AUC was determined as a comprehensive index of diagnostic value, with AUC <0.7, 0.7 ≤ AUC < 0.9, and AUC ≥0.9 indicating low, moderate, and high predictive value, respectively. Heterogeneity was explored using Cochran’s Q statistic and I^2^ statistic. I^2^ values of <30%, 30–50%, and >50% indicated low, moderate, and marked heterogeneity, respectively. A random-effects model was used when I^2^ >50% and a *p*-value < 0.1, otherwise, a fixed-effects model was applied. To ascertain the sources of heterogeneity, subgroup analysis and meta-regression were implemented. Meta-disc 1.4 (Ramón y Cajal Hospital, Madrid, Spain) was utilized to determine a threshold effect, which was indicated by a strong positive correlation (high Spearman correlation coefficient) between the logarithm of sensitivity and the logarithm of 1-specificity. Finally, sensitivity analyses were performed to check the stability of results by omitting one study at a time to eliminate factors affecting heterogeneity. Publication bias was analyzed using a Deeks funnel plot, with *p* < 0.05 indicating publication bias. This was mostly due to a preference for positive outcomes and selective outcome reporting.

## 3. Results

### 3.1 Study Selection

**Fig. 1.**
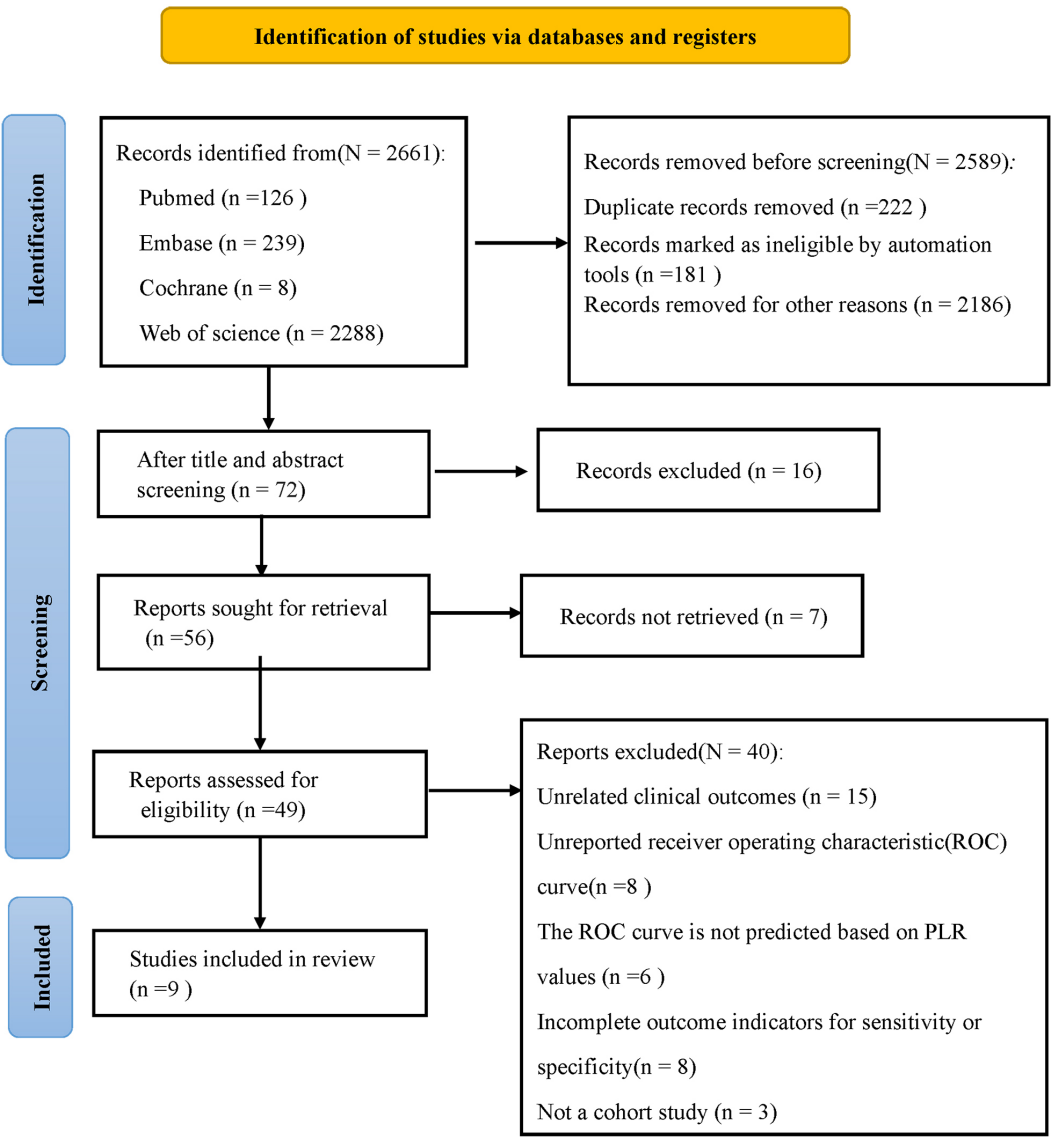
**Flowchart of database search and study inclusion**.

The study inclusion process is displayed in Fig. 1. An initial search identified 2661 relevant articles. Of these articles, 222 were excluded due to duplicates, 181 for unqualified literature types, and 2186 for irrelevant titles and abstracts. In the remaining 72 studies, 16 studies were excluded, and 7 articles were not accessible to the full text. 40 studies were excluded after the full-text review, among which 15 reported unrelated outcomes, 8 did not provide ROC curves, 14 had missing or unrelated sensitivity and specificity, and 3 were not cohort studies. Ultimately, 9 eligible studies [21, 22, 23, 24, 25, 26, 27, 28, 29] involving 7174 subjects were finally enrolled in the meta-analysis.

### 3.2 Baseline Characteristics 

Baseline characteristics and quality scores of the enrolled studies are displayed in Table 1 (Ref. [21, 22, 23, 24, 25, 26, 27, 28, 29]). These studies were published between 2014 and 2024, with 6 studies from China and 3 studies from Turkey. Regarding the experimental design, 4 studies were prospective cohort studies, and 5 were retrospective cohort studies. The PLR thresholds ranged from 107 to 225. The sample sizes ranged from 205 to 1938. The mean age of the patients ranged from 57 to 68 years. The male ratio across studies ranged from 0.66 to 0.81. Of note, 5 studies [23, 25, 26, 28, 29] reported in-hospital MACEs, 3 studies [21, 22, 24] reported long-term MACEs during follow-up, and 1 study [27] reported both in-hospital and long-term MACEs.

**Table 1.** **Baseline characteristics and quality scores of the included studies**.

Study year	Country	Study design	Sample size	Age	Gender (F/M)	Occurrence time	Follow-up time	Cut-off	Sensitivity	Specificity	TP	FP	FN	TN	NOS
Gao* et al*. [21]/2021	China	RC	1558	≥40	392/1166	Long-term MACEs	1142 days	225	0.31	0.87	20	192	43	1303	8
Wang and Peng [22]/2021	China	PC	387	68.06 ± 3.77	133/254	Long-term MACEs	6 months	112	0.84	0.52	81	140	15	151	8
Sheng* et al*. [23]/2021	China	PC	205	64.83 ± 10.77	67/138	In-hospital MACEs	-	107	0.74	0.48	14	97	5	89	7
Toprak *et al*. [24]/2015	Turkey	RC	304	59.80 ± 10.10	58/246	Long-term MACEs	22-26 months	201	0.70	0.66	48	80	21	155	8
Li* et al*. [25]/2021	China	RC	1001	59.55 ± 10.81	296/805	In-hospital MACEs	-	147	0.72	0.63	130	304	50	517	7
Wang* et al*. [26]/2023	China	PC	799	59.72 ± 10.69	156/643	In-hospital MACEs	30 days	173	0.65	0.73	44	197	24	534	7
Ozcan Cetin* et al*. [27]/2016	Turkey	PC	1938	59.73 ± 8.20	652/1286	In-hospital MACEs	-	143	0.70	0.68	95	581	40	1222	7
Ozcan Cetin* et al*. [27]/2016	Turkey	PC	1938	59.73 ± 8.20	652/1286	Long-term MACEs	31 months	147	0.72	0.70	210	488	81	1159	8
Ayça* et al*. [28]/2015	Turkey	RC	440	57.07 ± 7.85	146/294	In-hospital MACEs	-	137	0.63	0.67	52	118	31	239	7
Yang* et al*. [29]/2024	China	RC	542	61.08 ± 13.78	107/435	In-hospital MACEs	-	118	0.68	0.47	113	199	53	177	7

Note: RC, retrospective cohort study; PC, prospective observational study; Gender (F/M), ratio of female to male patients included in each original study; MACEs, major adverse cardiovascular events; Cut-off, PLR cut-off value; TP, true positive; FP, false positive; FN, false negative; TN, true negative; NOS, Newcastle-Ottawa Scale.

### 3.3 Quality Assessment

All studies scored between 7 and 8 on the NOS assessment, indicating high study quality. NOS scoring details are displayed in **Supplementary Table 2**.

### 3.4 Meta-Analysis

#### 3.4.1 Predictive Power of PLR for MACEs after PCI in ACS Patients

**Fig. 2.**
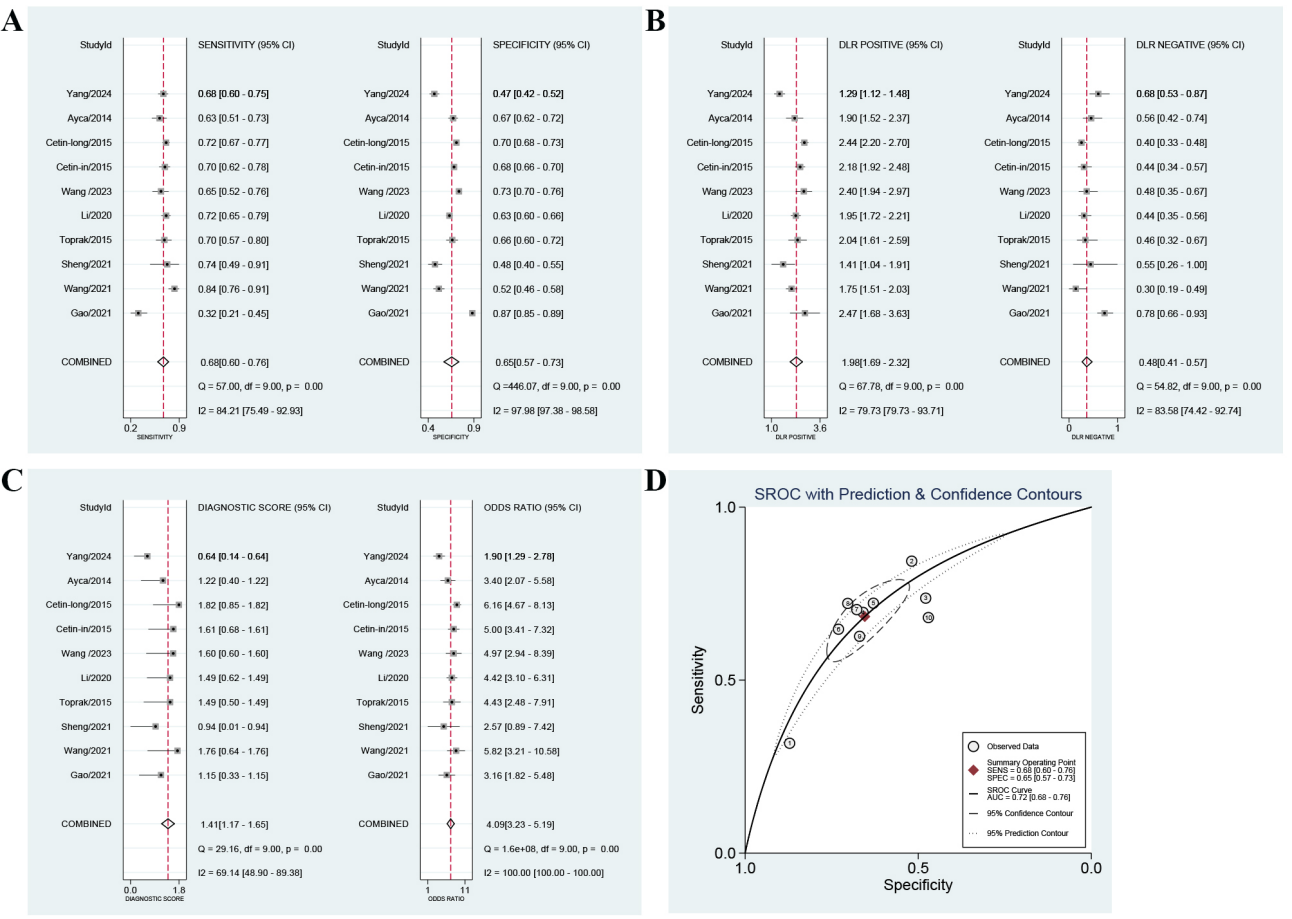
**Forest plots of the predictive power of platelet-to-lymphocyte ratio (PLR) for major adverse cardiovascular events (MACEs) after percutaneous coronary intervention (PCI) in acute coronary syndromes (ACS) patients**. (A) sensitivity and specificity, (B) positive likelihood ratio and negative likelihood ratio, (C) diagnostic odds ratio (DOR), and (D) summary receiver operating characteristic (SROC) curve. DLR, diagnostic likelihood ratio.

9 studies involving 7174 patients were enrolled. The forest plot manifested a combined value of 0.68 (95% CI, 0.60–0.76) for sensitivity and 0.65 (95% CI, 0.57–0.73) for specificity (Fig. 2A). The combined value was 1.98 (95% CI, 1.69–2.32) for positive LR, 0.48 (95% CI, 0.41–0.57) for negative LR (Fig. 2B), and 4.09 (95% CI, 3.23–5.19) for DOR (Fig. 2C). In addition, SROC analysis of high PLR for predicting the prognosis of ACS patients (Fig. 2D) showed a summary area under the curve (SAUC) of 0.72 (95% CI: 0.68–0.76), indicating that PLR had better predictive power for MACEs in ACS patients undergoing PCI.

#### 3.4.2 Subgroup Analysis and Meta-Regression

To further understand the predictive power of PLR for MACEs after PCI in ACS patients in the subgroups of different populations and different study designs, we performed subgroup analyses (Table 2) based on age (age ≤60 or age >60), sex ratio (≤30% of female patients or >30% of female patients), PLR cutoff value (PLR ≤150 or PLR >150), study type (prospective cohort study or retrospective cohort study), and time of MACEs (in-hospital or long-term) (Table 2). The results elicited that the combined value for sensitivity was higher in the age >60 subgroup (0.75 vs 0.64) and in the subgroup with >30% of female patients (0.72 vs 0.62). The subgroup of PLR cutoff ≤150 (0.73 vs 0.56) and the subgroup of prospective cohort studies (0.75 vs 0.61) had higher combined values for sensitivity. Notably, the combined sensitivity of the in-hospital MACEs subgroup was slightly higher than that of the long-term MACEs subgroup (0.69 vs 0.67).

**Table 2.** **Subgroup analysis of PLR predictive power**.

Project	Group	Sensitivity-analysis			Specificity-analysis		
		Nstudies	Sensitivity	*p*	Nstudies	Specificity	*p*
Age	Average age ≤60	7	0.64 [0.56–0.73]	0.63	7	0.72 [0.66–0.77]	0.00
	Average age >60	3	0.75 [0.64–0.87]		3	0.49 [0.38–0.59]	
Gender	F/M+F ≤30%	5	0.62 [0.51–0.72]	0.71	5	0.69 [0.59–0.79]	0.05
	F/M+F >30%	5	0.75 [0.66–0.83]		5	0.62 [0.50–0.73]	
Cut-off	Cut-off ≤150	7	0.73 [0.66–0.80]	0.01	7	0.60 [0.52–0.68]	0.89
	Cut-off >150	3	0.56 [0.42–0.69]		3	0.77 [0.68–0.86]	
Research type	PC	5	0.75 [0.67–0.84]	0.00	5	0.63 [0.52–0.74]	0.44
	RC	5	0.61 [0.51–0.72]		5	0.68 [0.57–0.78]	
MACEs occurrence time	In-hospital MACEs	6	0.69 [0.59–0.79]	0.15	6	0.62 [0.52–0.72]	0.61
	Long-term MACEs	4	0.67 [0.55–0.80]		4	0.71 [0.60–0.82]	

The combined specificity was higher in the aged ≤60 subgroup (0.72 vs 0.49) and the subgroup with ≤30% female patients (0.69 vs 0.62). The subgroup of PLR >150 (0.77 vs 0.60) and the subgroup of retrospective cohort studies (0.68 vs 0.63) had higher combined specificity. The long-term MACEs subgroup had a slightly higher combined specificity than the in-hospital MACEs subgroup (0.71 vs 0.62).

**Fig. 3.**
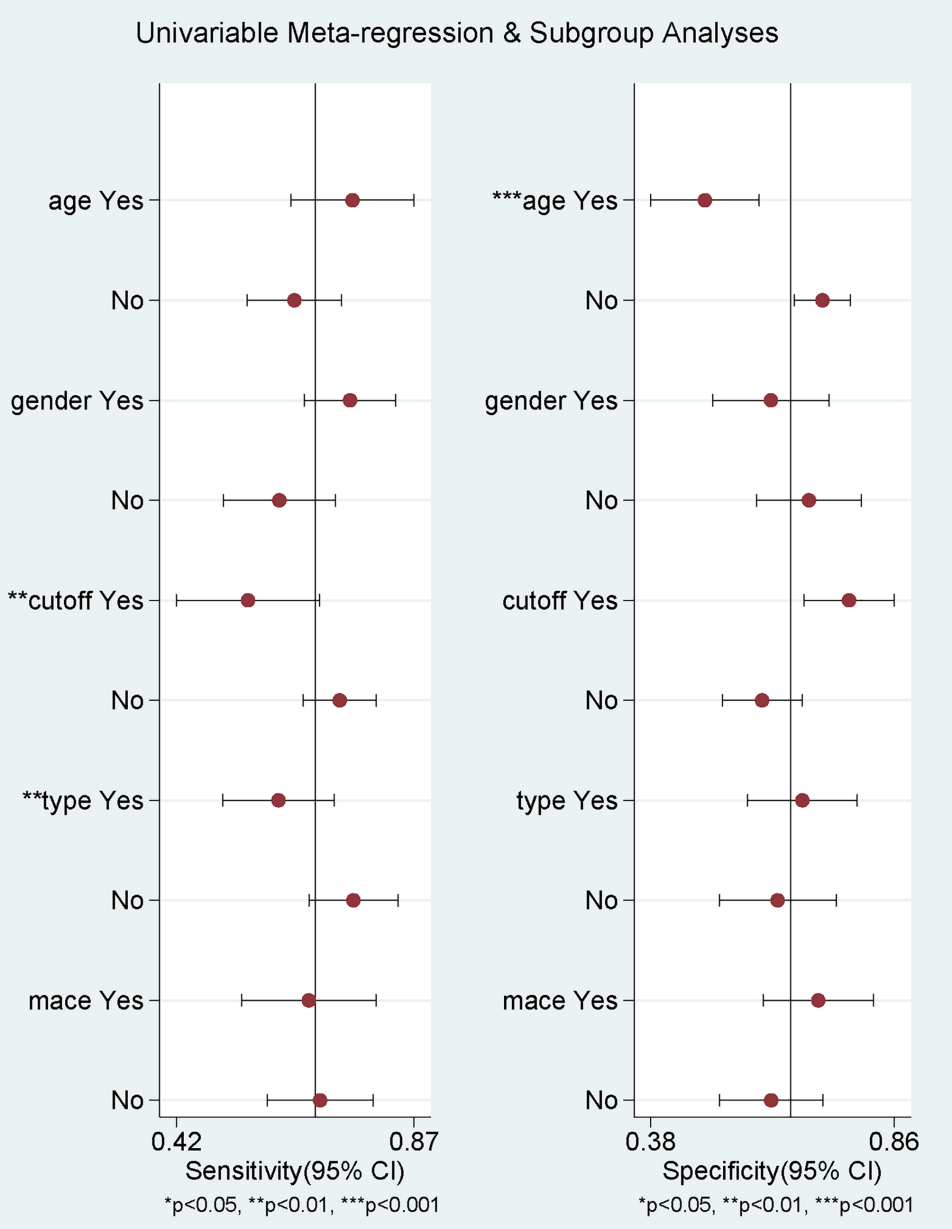
**Meta-regression analysis of the source of heterogeneity**.

To further unveil the source of heterogeneity, we performed meta-regression (Fig. 3). For sensitivity, study type (*p* < 0.01) and PLR cutoff (*p* < 0.01) might be the sources of heterogeneity. For specificity, mean patient age (*p* < 0.001) and sex ratio (*p* = 0.05) might be the sources of heterogeneity.

#### 3.4.3 Threshold Effect

Spearman’s correlation coefficient showed a correlation coefficient of 0.564 and a *p*-value of 0.09, indicating no threshold effect.

#### 3.4.4 Sensitivity Analysis

**Fig. 4.**
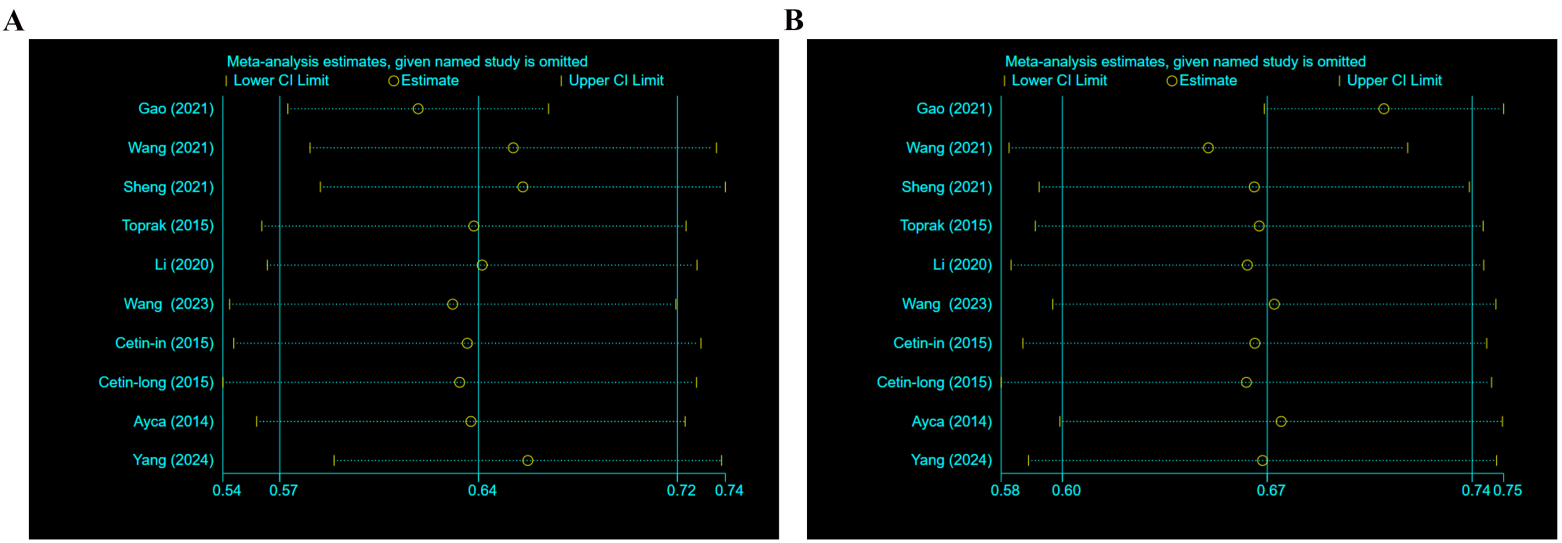
**Sensitivity analysis**. (A) Sensitivity analysis of specificity. (B) Sensitivity analysis of sensitivity.

The sensitivity analysis proved the robustness of the results (Fig. 4A,B).

#### 3.4.5 Publication Bias

**Fig. 5.**
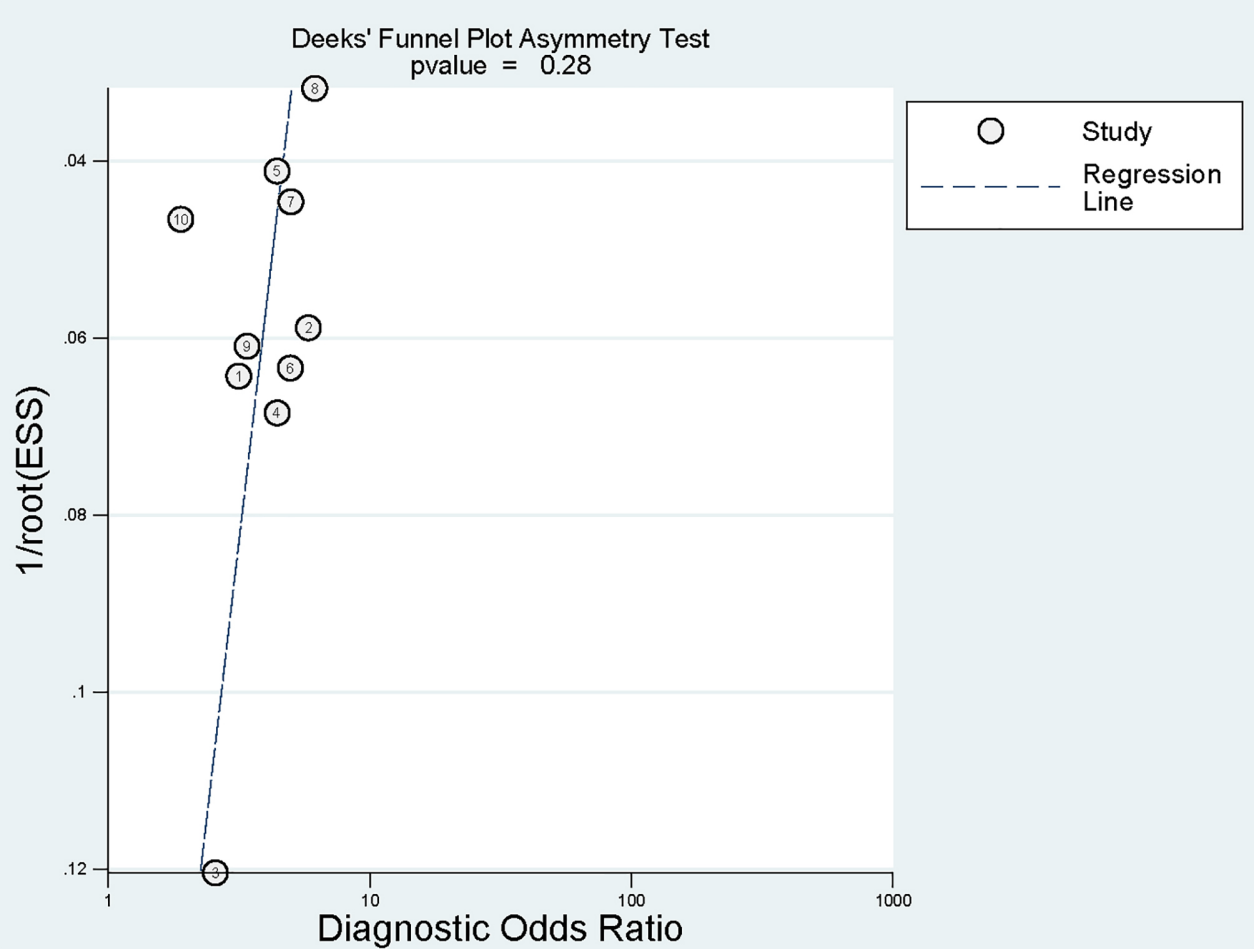
**Deeks funnel plot for publication bias**. ESS, effective sample size.

Deeks funnel plot implied no significant publication bias (*p* = 0.28) (Fig. 5).

## 4. Discussion

ACS patients are at high risk for MACEs and poor prognoses after PCI and are prone to coronary revascularization, heart failure, cardiovascular death, myocardial infarction, and stroke [6, 7, 8, 9, 10]. Therefore, early prediction and identification of MACEs after PCI in ACS patients appears to be critical. This is the first meta-analysis (including 9 papers and 7174 subjects) on the predictive power of PLR for MACEs after PCI in ACS patients. Our results illustrated that PLR had favorable performance in predicting MACEs after PCI in ACS patients, as evidenced by the high combined sensitivity, specificity, and SAUC values. Furthermore, PLR in all ACS patients undergoing PCI had greater sensitivity and a better ability of risk identification in patients aged >60 years and the subgroup with a higher proportion of women and was also more sensitive to in-hospital MACEs.

The results noted that PLR was a favorable biological predictor of MACEs after PCI in ACS patients. Similarly, previous finding [30] highlighted that higher PLR before PCI was an independent predictor of long-term adverse clinical outcomes in patients with NSTEMI and patients with unstable angina. Oylumlu *et al*. [31] explored the link between PLR and in-hospital mortality in ACS patients and uncovered that with 142 as the cutoff point, PLR predicted in-hospital mortality with 69% sensitivity and 63% specificity, suggesting that PLR can be viewed as a predictor of in-hospital mortality in ACS patients. Another study [32] reported that PLR ≥116 had 71% sensitivity and 66% specificity in forecasting moderate-to-high Synergy Between PCI With Taxus and Cardiac Surgery (SYNTAX) scores for MACEs and mortality in ACS patients.

The potential mechanisms of PLR in forecasting MACEs after PCI in ACS patients are complex. PLR integrates two key parameters: high platelet counts and low lymphocyte counts. These two parameters are correlated with MACEs in ACS patients [33, 34, 35]. On the one hand, platelets are involved in atherosclerosis and thrombosis after destabilization of atherosclerotic plaques, which is closely related to MACEs in ACS patients after surgery [36, 37]. An elevated platelet count reflects an excessive inflammatory state. As a biomarker of systemic inflammatory responses, pathologically elevated platelet count is significantly and positively correlated with excessive inflammatory status. In the context of inflammatory cytokine storm, pro-inflammatory mediators such as interleukin-6, C-reactive protein, and tumor necrosis factor-α induce matrix metalloproteinases (MMPs) in vascular endothelial cells, especially MMP-2 and MMP-9, leading to the collagen matrix degradation of NF-κB signaling and the structural rupture of vulnerable plaques [38, 39, 40]. After plaque rupture, exposure of the subendothelial procoagulant matrix initiates the tissue factor-dependent coagulation cascade, prompting the assembly of the prothrombinase complex and accelerating thrombin generation. This enzymatic process catalyzes the conversion of fibrinogen into fibrin to form a three-dimensional network structure and also mediates platelet activation through protease-activated receptors. Activated platelets express P-selectin and glycoprotein IIb/IIIa (GPIIb/IIIa) receptors, which bind to the damaged endothelium through von Willebrand factor (vWF)-mediated adhesion mechanisms. Under the action of secondary agonists such as adenosine diphosphate (ADP) and thromboxane A2 (TXA2), irreversible aggregation occurs, eventually forming a pro-thrombotic microenvironment [41, 42, 43]. Additionally, megakaryocytes promote platelet secretion under the stimulation of inflammatory mediators, resulting in a prothrombotic state [24, 44]. On the other hand, platelets can release thromboxane and other mediators to promote monocyte adhesion and migration, which aggravates inflammation and weakens plaque stability, promoting the progression of atherosclerosis and increasing the risk of MACEs [45, 46, 47, 48]. High platelet counts may suggest that platelet-rich thrombi are more likely to form on atherosclerotic plaques, which may lead to no flow after PCI, resulting in poor prognoses [49, 50]. In terms of the mechanisms of lymphocytes in MACEs, several studies have reported that decreased lymphocyte counts are associated with adverse outcomes in CVDs [36, 51, 52]. Lymphocytes are crucial in myocardial healing after AMI and in maintaining the stability of atherosclerotic plaques in ACS patients [36, 51, 53]. In addition, the physiological stress response of patients elevates cortisol and suppresses the immune response, which lowers lymphocyte counts and results in poor clinical outcomes in ACS patients [32, 54]. The above demonstrates that PLR can predict postoperative outcomes in ACS patients. More attention should be paid to ACS patients with high preoperative PLR, which is helpful to recognize and predict the risk of postoperative MACEs as early as possible and to give appropriate management interventions.

Our study evinced that PLR was more effective in forecasting MACEs after PCI in female ACS patients. Previous studies have found differences in platelet counts by sex (higher platelet count values in women than in men), and enhanced platelet aggregation may contribute to an increased susceptibility to plaque rupture and consequent thrombosis, leading to an elevated risk of MACEs [55, 56]. Proteinase-activated receptor-1 (PAR-1)-mediated platelet reactivity is notably higher in women [57, 58]. In addition, women are more sensitive to platelet aggregation stimuli than men [57], which may accelerate thrombosis after plaque rupture and lead to adverse outcomes. Higher platelet counts, platelet reactivity, and sensitivity in women suggest that PLR values are more effective in forecasting MACEs after surgery in female patients. Interestingly, sex specificity is also present in platelet activation. More pronounced leukocyte-platelet aggregates (LPA) are observed in females in response to thrombin receptor-activating peptide-6 (TRAP-6, agonist of PAR-1) or adenosine diphosphate [58]. LPA formation is a stable marker for platelet activation. High LPA may suggest that female patients have strong platelet activation and leukocyte-platelet interactions, leading to a prothrombotic state. It is also associated with postoperative adverse events in female patients since thrombosis and vasoconstriction induce no vascular reflux [56, 59]. The PLR value, which combines the predictive values of platelet and lymphocyte counts and reflects their interactions to some extent, may be a favorable predictor of MACEs in female patients.

Furthermore, PLR had better predictive power in patients >60 years. With increasing age, platelets show increased reactivity and sensitivity to aggregation stimuli, resulting in an enhanced prethrombotic state. Older patients may also suffer from a chronic inflammatory state due to the presence of cumulative diseases and the ongoing process of tissue damage and repair [16]. Repeated activation in chronic inflammatory states may suppress lymphocyte counts, and lymphocytes also play a key role in plaque stability [16, 60]. Besides, dysregulation of pro-inflammatory mediators and upregulation of cytokines, chemokines, and their receptors due to the chronic inflammatory state would exaggerate the inflammatory response, enhance platelet reactivity, and contribute to thrombosis and adverse events in older ACS patients after PCI. Higher PLR reflects increased platelet counts and activity and reduced lymphocytes, which may facilitate the rupture of vulnerable plaques and the re-formation of thrombi, leading to MACEs. A previous study reported substantially higher PLR in the older group, which predicted cardiac rupture, acute heart failure, and total adverse events (specificity 72%; sensitivity 63%) [25]. In another study [16], PLR predicted cardiovascular mortality in ACS patients >65 years, whereas this prediction ability was not observed in the subgroup <65 years. These results confirm that PLR values have better predictive power for MACEs in the older ACS population.

Preoperative PLR values reflect the state of inflammatory responses at blood collection and for a short period, and therefore it may be more sensitive for forecasting short-term in-hospital MACEs. One study showed that postoperative PLR value in ACS patients was first increased and then decreased, suggesting elevated inflammatory markers in ACS patients after PCI [23]. The possible reason is that PCI further promotes inflammatory responses, which are often associated with the frequency of intracoronary treatments, the number and length of stent placements, maximum pressure of intraoperative balloon dilatation, and procedure time [11, 12, 23]. This can also lead to high sensitivity of PLR for predicting in-hospital MACEs. However, long-term postoperative conditions are difficult to control and may be affected by postoperative medications, poor lifestyle habits, and gradual disease progression. Therefore, the combined sensitivity of PLR for forecasting in-hospital MACEs is slightly higher than that for long-term MACEs, suggesting that PLR is more sensitive for forecasting in-hospital MACEs in ACS patients undergoing PCI.

Inflammation is a potential causal factor for CAD and may contribute to adverse outcomes in ACS patients [61]. One study [62] identified five lymphocyte-based inflammatory markers (PLR, neutrophil-lymphocyte ratio [NLR], monocyte-lymphocyte ratio [MLR], systemic immune inflammation index [SII], and systemic inflammatory response index [SIRI]) as independent predictors of MACEs. These markers highlighted the limitations of PLR. Another study demonstrated greater sensitivity of the PLR-NLR combination index in predicting acute myocardial infarction than PLR or NLR alone [15]. Additionally, Zhou* et al*. [63] found a positive association between the Global Registry of Acute Coronary Events (GRACE) risk score and PLR, suggesting that the combination of PLR and GRACE score was more effective in predicting CVD events in ACS patients. These existing studies provide references for clinical applications of PLR and guide further research.

This meta-analysis has several limitations. First, it included a relatively small number of studies (9) from China and Turkey, which may limit the generalizability of the findings and introduce potential bias. Second, the PLR thresholds in the meta-analysis exhibited significant heterogeneity, possibly attributed to the true effect of the included populations or differences in the detection tools used, which we are unable to address here. Finally, we focused solely on the predictive power of the PLR value before PCI for MACEs in ACS patients and failed to further explore the potential predictive value of postoperative PLR and dynamic changes in PLR, which should be considered as future research directions.

## 5. Conclusion

This meta-analysis illustrates that preoperative PLR values have favorable predictive power for in-hospital and long-term MACEs in ACS patients treated with PCI. PLR shows higher sensitivity and better risk identification in patients >60 years and the subgroup with a higher proportion of women and was also more sensitive to in-hospital MACEs. As a simple and readily available biomarker, PLR can be applied along with other tools or inflammatory indicators to guide prognostic assessment and follow-up for ACS patients undergoing PCI. Notably, the potential predictive value of both postoperative PLR and dynamic changes in PLR should be explored in future studies. Meanwhile, further exploration of the potential impact of PLR thresholds on its predictive performance could improve the applicability of PLR in different patient populations.

## Availability of Data and Materials

The data underlying this article are available in the article and its online supplementary material.

## Supplementary Material

Supplementary material associated with this article can be found, in the online version, at https://doi.org/10.31083/RCM27942.
