# Supplementary material for: Platelet-To-Lymphocyte Ratio Efficiency in Predicting Major Adverse Cardiovascular Events After Percutaneous Coronary Intervention in Acute Coronary Syndromes: A Meta-Analysis
Source: Rev Cardiovasc Med. 2025 May 21;26(5):27942. doi: 10.31083/RCM27942 (PMC12135647; doi:10.31083/RCM27942)
Supplement: Supplementary file 1 [file 2153-8174-26-5-27942-s1.zip › Supplementary Materials.docx]

**Table S1:** Search strategy

**Cochrane**

ID Search Hits

#1 MeSH descriptor: [Blood Platelets] explode all trees 2543

#2 (Thrombocyte*):ti,ab,kw OR (Platelet*):ti,ab,kw 39680

#3 #1 OR #2 39680

#4 MeSH descriptor: [Lymphocytes] explode all trees 7259

#5 (Lymphocyte):ti,ab,kw OR (Lymphoid Cell*):ti,ab,kw OR (immune competent cell):ti,ab,kw OR (immunocyte):ti,ab,kw OR (lymph cell):ti,ab,kw 26642

#6 #4 OR #5 29412

#7 MeSH descriptor: [Acute Coronary Syndrome] explode all trees 3192

#8 (Acute Coronary Syndrome*):ti,ab,kw OR (unstable angina pectoris):ti,ab,kw OR (myocardial infarction):ti,ab,kw 44145

#9 #7 OR #8 44145

#10 MeSH descriptor: [Percutaneous Coronary Intervention] explode all trees 9038

#11 (Percutaneous Coronary Intervention*):ti,ab,kw OR (Percutaneous Coronary Revascularization*):ti,ab,kw 14734

#12 #10 OR #11 16911

#13 #3 AND #6 AND #9 AND #12 8

**Pubmed**

| Search number | Query | Search Details | Results |
| --- | --- | --- | --- |
| 13 | ((((Blood Platelets[MeSH Terms]) OR ((Thrombocyte*[Title/Abstract]) OR (Platelet*[Title/Abstract]))) AND ((Lymphocytes[MeSH Terms]) OR (((((Lymphocyte[Title/Abstract]) OR (Lymphoid Cell*[Title/Abstract])) OR (immune competent cell[Title/Abstract])) OR (immunocyte[Title/Abstract])) OR (lymph cell[Title/Abstract])))) AND ((Acute Coronary Syndrome[MeSH Terms]) OR (((Acute Coronary Syndrome*[Title/Abstract]) OR (unstable angina pectoris[Title/Abstract])) OR (myocardial infarction[Title/Abstract])))) AND ((Percutaneous Coronary Intervention[MeSH Terms]) OR ((Percutaneous Coronary Intervention*[Title/Abstract]) OR (Percutaneous Coronary Revascularization*[Title/Abstract]))) | ("blood platelets"[MeSH Terms] OR ("thrombocyte*"[Title/Abstract] OR "platelet*"[Title/Abstract])) AND ("lymphocytes"[MeSH Terms] OR ("Lymphocyte"[Title/Abstract] OR "lymphoid cell*"[Title/Abstract] OR "immune competent cell"[Title/Abstract] OR "immunocyte"[Title/Abstract] OR "lymph cell"[Title/Abstract])) AND ("acute coronary syndrome"[MeSH Terms] OR ("acute coronary syndrome*"[Title/Abstract] OR "unstable angina pectoris"[Title/Abstract] OR "myocardial infarction"[Title/Abstract])) AND ("percutaneous coronary intervention"[MeSH Terms] OR ("percutaneous coronary intervention*"[Title/Abstract] OR "percutaneous coronary revascularization*"[Title/Abstract])) | 126 |
| 12 | (Percutaneous Coronary Intervention[MeSH Terms]) OR ((Percutaneous Coronary Intervention*[Title/Abstract]) OR (Percutaneous Coronary Revascularization*[Title/Abstract])) | "percutaneous coronary intervention"[MeSH Terms] OR "percutaneous coronary intervention*"[Title/Abstract] OR "percutaneous coronary revascularization*"[Title/Abstract] | 86,699 |
| 11 | (Percutaneous Coronary Intervention*[Title/Abstract]) OR (Percutaneous Coronary Revascularization*[Title/Abstract]) | "percutaneous coronary intervention*"[Title/Abstract] OR "percutaneous coronary revascularization*"[Title/Abstract] | 50,979 |
| 10 | Percutaneous Coronary Intervention[MeSH Terms] | "percutaneous coronary intervention"[MeSH Terms] | 68,657 |
| 9 | (Acute Coronary Syndrome[MeSH Terms]) OR (((Acute Coronary Syndrome*[Title/Abstract]) OR (unstable angina pectoris[Title/Abstract])) OR (myocardial infarction[Title/Abstract])) | "acute coronary syndrome"[MeSH Terms] OR "acute coronary syndrome*"[Title/Abstract] OR "unstable angina pectoris"[Title/Abstract] OR "myocardial infarction"[Title/Abstract] | 256,424 |
| 8 | ((Acute Coronary Syndrome*[Title/Abstract]) OR (unstable angina pectoris[Title/Abstract])) OR (myocardial infarction[Title/Abstract]) | "acute coronary syndrome*"[Title/Abstract] OR "unstable angina pectoris"[Title/Abstract] OR "myocardial infarction"[Title/Abstract] | 253,759 |
| 7 | Acute Coronary Syndrome[MeSH Terms] | "acute coronary syndrome"[MeSH Terms] | 21,638 |
| 6 | (Lymphocytes[MeSH Terms]) OR (((((Lymphocyte[Title/Abstract]) OR (Lymphoid Cell*[Title/Abstract])) OR (immune competent cell[Title/Abstract])) OR (immunocyte[Title/Abstract])) OR (lymph cell[Title/Abstract])) | "lymphocytes"[MeSH Terms] OR "Lymphocyte"[Title/Abstract] OR "lymphoid cell*"[Title/Abstract] OR "immune competent cell"[Title/Abstract] OR "immunocyte"[Title/Abstract] OR "lymph cell"[Title/Abstract] | 689,731 |
| 5 | ((((Lymphocyte[Title/Abstract]) OR (Lymphoid Cell*[Title/Abstract])) OR (immune competent cell[Title/Abstract])) OR (immunocyte[Title/Abstract])) OR (lymph cell[Title/Abstract]) | "Lymphocyte"[Title/Abstract] OR "lymphoid cell*"[Title/Abstract] OR "immune competent cell"[Title/Abstract] OR "immunocyte"[Title/Abstract] OR "lymph cell"[Title/Abstract] | 214,387 |
| 4 | Lymphocytes[MeSH Terms] | "lymphocytes"[MeSH Terms] | 594,223 |
| 3 | (Blood Platelets[MeSH Terms]) OR ((Thrombocyte*[Title/Abstract]) OR (Platelet*[Title/Abstract])) | "blood platelets"[MeSH Terms] OR "thrombocyte*"[Title/Abstract] OR "platelet*"[Title/Abstract] | 283,239 |
| 2 | (Thrombocyte*[Title/Abstract]) OR (Platelet*[Title/Abstract]) | "thrombocyte*"[Title/Abstract] OR "platelet*"[Title/Abstract] | 271,871 |
| 1 | Blood Platelets[MeSH Terms] | "blood platelets"[MeSH Terms] | 84,483 |

**Embase**

| No. | Query | Results |
| --- | --- | --- |
| #13 | #3 AND #6 AND #9 AND #12 | 239 |
| #12 | #10 OR #11 | 147000 |
| #11 | 'percutaneous coronary intervention*':ab,ti OR 'percutaneous coronary revascularization*':ab,ti | 79901 |
| #10 | 'percutaneous coronary intervention'/exp | 139815 |
| #9 | #7 OR #8 | 406265 |
| #8 | 'acute coronary syndrome*':ab,ti OR 'unstable angina pectoris':ab,ti OR 'myocardial infarction':ab,ti | 385795 |
| #7 | 'acute coronary syndrome'/exp | 83013 |
| #6 | #4 OR #5 | 1261123 |
| #5 | lymphocyte:ab,ti OR 'lymphoid cell*':ab,ti OR 'immune competent cell':ab,ti OR immunocyte:ab,ti OR 'lymph cell':ab,ti | 280599 |
| #4 | 'lymphocyte'/exp | 1141823 |
| #3 | #1 OR #2 | 425460 |
| #2 | thrombocyte*:ab,ti OR platelet*:ab,ti | 401085 |
| #1 | 'thrombocyte'/exp | 142130 |

**Web of Science**

1: Blood Platelets (Topic) OR Thrombocyte* (Topic) OR Platelet* (Topic) and Preprint Citation Index (Exclude – Database)

Running Date: Fri Feb 7 2025 14:42:28 GMT+0800 (China Standard Time) Retrieved result: 495604

2: Lymphocyte* (Topic) OR Lymphoid Cell* (Topic) OR immune competent cell (Topic) OR immunocyte (Topic) OR lymph cell (Topic) and Preprint Citation Index (Exclude – Database)

Running Date: Fri Feb 7 2025 14:44:30 GMT+0800 (China Standard Time) Retrieved result:3253823

3: Acute Coronary Syndrome* (Topic) OR unstable angina pectoris (Topic) OR myocardial infarction (Topic) and Preprint Citation Index (Exclude – Database)

Running Date: Fri Feb 7 2025 14:45:29 GMT+0800 (China Standard Time) Retrieved result: 546625

4: Percutaneous Coronary Intervention* (Topic) OR Percutaneous Coronary Revascularization* (Topic) and Preprint Citation Index (Exclude – Database)

Running Date: Fri Feb 7 2025 14:46:00 GMT+0800 (China Standard Time) Retrieved result: 108413

5:#1 AND #2 AND #3 AND #4 AND #5 and Preprint Citation Index (Exclude – Database)

Running Date: Fri Feb 7 2025 14:46:53 GMT+0800 (China Standard Time) Retrieved result: 2288

**Table S2:** NOS scoring details

| **TITLE** | **SELECTION** | | | | **COMPAR-ABILITY** | **OUTCOME** | | | **Num** |
| --- | --- | --- | --- | --- | --- | --- | --- | --- | --- |
|  | **Representativeness of Exposed Cohort** | **Selection of the Non-Exposed Cohort** | **Ascertainment of Exposure** | **Outcome of Interest Was Not Present at Start of Study** |  | **Assessment of Outcome** | **Follow-Up Long Enough** | **Adequacy of Follow Up** |  |
| Gao et al.  [21]/2021 | 1 | 1 | 1 | 1 | 1 | 1 | 1 | 1 | 8 |
| Wang et al.  [22]/2021 | 1 | 1 | 1 | 1 | 1 | 1 | 1 | 1 | 8 |
| Sheng et al.  [23]/2021 | 1 | 1 | 1 | 1 | 1 | 1 | 0 | 1 | 7 |
| Toprak et al.  [24]/2015 | 1 | 1 | 1 | 1 | 1 | 1 | 1 | 1 | 8 |
| Li et al.  [25]/2020 | 1 | 1 | 1 | 1 | 1 | 1 | 0 | 1 | 7 |
| Wang et al.  [26]/2023 | 1 | 1 | 1 | 1 | 1 | 1 | 0 | 1 | 7 |
| Cetin et al.  [27]/2015 | 1 | 1 | 1 | 1 | 1 | 1 | 0 | 1 | 7 |
| Cetin et al.  [27]/2015 | 1 | 1 | 1 | 1 | 1 | 1 | 1 | 1 | 8 |
| Ayca et al.  [28]/2014 | 1 | 1 | 1 | 1 | 1 | 1 | 0 | 1 | 7 |
| Yang et al.  [29]/2024 | 1 | 1 | 1 | 1 | 1 | 1 | 0 | 1 | 7 |
